# Assessment of autosomal and male DNA extracted from casework samples using Casework Direct Kit, Custom and Maxwell 16 System DNA IQ Casework Pro Kit for autosomal-STR and Y-STR profiling

**DOI:** 10.1038/s41598-019-51154-4

**Published:** 2019-10-10

**Authors:** Hashom Mohd Hakim, Hussein Omar Khan, Siti Afifah Ismail, Shahrizad Ayob, Japareng Lalung, Edward Abban Kofi, Geoffrey Keith Chambers, Hisham Atan Edinur

**Affiliations:** 1 DNA Databank Division (D13), Criminal Investigation Department, Royal Malaysia Police, 43200 Cheras, Selangor Malaysia; 2 School of Industrial Technology, Universiti Sains Malaysia, 11800 Pulau Pinang, Malaysia; 3 Forensic Science Programme, School of Health Sciences, Universiti Sains Malaysia, Health Campus, 16150 Kubang Kerian, Kelantan Malaysia; 4 Forensic Science Laboratory, Criminal Investigation Department, Ghana Police Service, P.O. Box 505, Accra, Ghana; 5 School of Biological Sciences, Victoria University of Wellington, P.O. Box 600, Wellington, New Zealand; 6 Institute of Tropical Biodiversity and Sustainable Development, Universiti Malaysia Terengganu, 21030 Kuala Nerus, Terengganu Malaysia; 7 Environmental Futures Research Institute, Griffith University, Nathan, Queensland 4111 Australia

**Keywords:** Genetic markers, Genetic techniques

## Abstract

Short repetitive regions in autosomal and Y chromosomes known as short tandem repeats (STRs) are currently used for DNA profiling in crime investigations. However, DNA profiling requires a sufficient quality and quantity of DNA template, which is often not obtained from trace evidence or degraded biological samples collected at the scene of a crime. Here, we assessed autosomal and male DNA components extracted from crime scene and mock casework samples using the Casework Direct Kit, Custom and compared the results against those obtained by extraction of matching samples using well-established Maxwell 16 System DNA IQ Casework Pro Kit. The quantity and quality of extracted DNA obtained using both Casework Direct Kit, Custom and Maxwell 16 System DNA IQ Casework Pro Kit were analyzed using PowerQuant Systems followed by autosomal and Y-chromosome STR profiling using GlobalFiler Express PCR Amplification Kit and PowerPlex Y23 System, respectively. Our results showed that the Casework Direct Kit and Maxwell 16 DNA IQ Casework Pro Kit have more or less equal capacity to extract inhibitor free DNA, but that the latter produces slightly better quality and more DNA template and subsequently higher numbers of STR allele calls for autosomal and Y-STR analyses. Nonetheless, the Casework Direct Kit, Custom is the quicker and cheaper option for extraction of good, clean DNA from high content material and might best be used for extraction of reference samples. Such reference DNA samples typically come from buccal swabs or freshly drawn blood. So, in general, they can confidently be expected to have a high nucleic acid content and to be inhibitor-free.

## Introduction

The last few decades have seen many significant advances in the field of forensic science, including greatly improved methods for individual identification. Several short repetitive regions in human genome, known as short tandem repeats (STRs), are currently used to identify individuals in crime investigations. However, to work effectively DNA profiling techniques require a sufficient quality and quantity of DNA template. Such material is often not obtained from trace evidence or degraded biological samples collected at the scene of a crime. In addition, increasing number of forensic DNA casework samples submitted to crime laboratories may delay particular criminal investigations due to limited number of experienced analysts who may be available together with queues due to prolonged sample screening and processing time^1^. Therefore, a modern forensic DNA service should be equipped with a reliable machine platform capable of avoiding human errors, reducing crossover contamination, and improving turnaround time. These requirements have led to the development of rapid automated platforms for DNA extraction, amplification^2–4^ and quantitation^5–11^. The manufacturer of these platforms claim that they enable accurate quantitation of genomic and male DNA plus improve the quality of extracted nucleic acid^12^.

One of the most commonly used methods to extract and purify DNA from casework samples is magnetic bead-based purification of nucleic acids. This technique requires multiple washing procedures to remove PCR inhibitors and not all of the DNA can be recovered from these washing steps. Promega Corporation has developed a new system, the Casework Direct Kit, Custom that does not need repeated washing steps in extracting the nucleic acids. It contains only casework extraction buffer and a reducing agent, 1-thioglycerol, a combination that is designed to rapidly produce DNA lysate from casework samples. This DNA lysate can be directly used for STR amplification if the quality and quantity of extracted DNA reach the minimum standard set by the individual DNA forensic laboratory as determined through quantitative PCR (qPCR) analysis. The kit has been proposed to be particularly useful for screening sexual assault and ‘touch’ (aka skin contact) DNA samples containing low quantities of male DNA^12,13^, because it is able to extract the available DNA and no washing steps are required as compared with magnetic bead-based purification methods. Thus, the Casework Direct Kit, Custom is said to be useful for rapid extraction of casework samples and able to produce amplification-ready lysates quickly with minimum ‘hands on’ time^12^.

In this study, we assessed autosomal and male DNA components extracted from touch DNA, blood and saliva stained samples using the Casework Direct Kit, Custom (Promega Corporation, Madison, USA) and compared the results against those obtained by extraction of matching samples using well-established Maxwell 16 System DNA IQ Casework Pro Kit^14,15^. The Maxwell 16 System DNA IQ Casework Pro Kit utilize paramagnetic beads to capture DNA followed by a number of washing steps. The DNA template is normally eluted in a final volume of 50 µl elution buffer. This system has been used on a wide range of samples^16^ and shown to be reliable for casework-type materials. In contrast, the performance of the Casework Direct Kit, Custom has never been systematically tested using real casework samples which often contain limited amounts of low quality of DNA. Thus, quality and quantity of DNA extracted using the Casework Direct Kit, Custom can be confidently compared with those obtained from the well-tested Maxwell 16 System DNA IQ Casework Pro Kit. Our casework samples were collected by Crime Scene Investigation Forensic Unit (D10), the Selangor State Police Contingent, Malaysia from several crime scenes, while mock and a female buccal swab samples were prepared in-house. The quantity and quality of extracted DNA obtained using both Casework Direct Kit, Custom and Maxwell 16 System DNA IQ Casework Pro Kit were then compared and analyzed using PowerQuant Systems (Promega Corporation, Madison, USA) followed by autosomal and Y-chromosome STR profiling using GlobalFiler Express PCR Amplification Kit (Applied Biosystem) and PowerPlex Y23 System (Promega Corporation, Madison, USA), respectively.

## Results and Discussion

**Figure 1:**
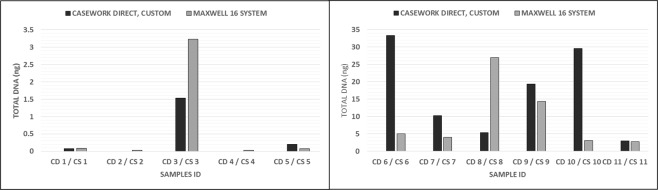
Amount of genomic extracted using Casework Direct Kit, Custom and Maxwell 16 System DNA IQ Casework Pro Kit. We obtained high DNA concentration (7712.76 ng/ul and 945.1 ng/ul for Casework Direct Kit, Custom and Maxwell 16 System DNA IQ Casework Pro Kit, respectively) for the female reference samples (i.e. CD12/CS 12). Data for these reference samples were not shown in the graph as it will distort value for other data points.

**Figure 2:**
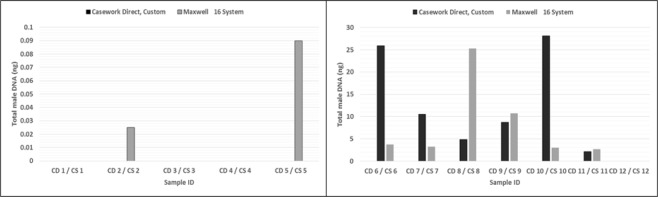
Amount of male DNA extracted using Casework Direct Kit, Custom and Maxwell 16 System DNA IQ Casework Pro Kit.

**Figure 3:**
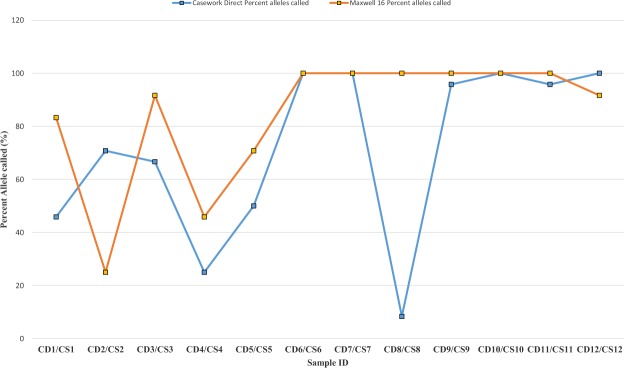
Percentages of autosomal STR allele calls for DNA extracted using Casework Direct, Custom Kit and Maxwell16 System DNA IQ Casework Pro Kit.

**Figure 4:**
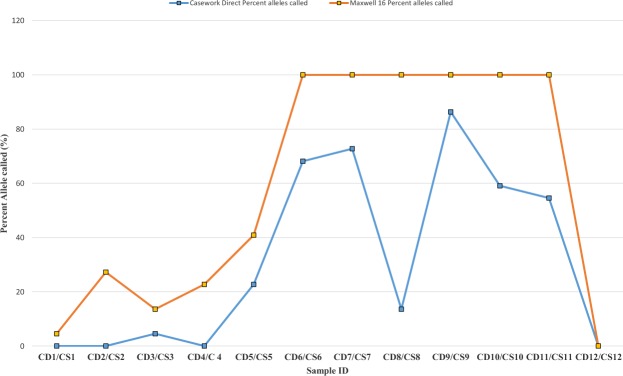
Percentages of Y chromosome STR allele calls for male DNA extracted using Casework Direct, Custom Kit and Maxwell 16 System DNA IQ Casework Pro kit.

**Figure 5:**
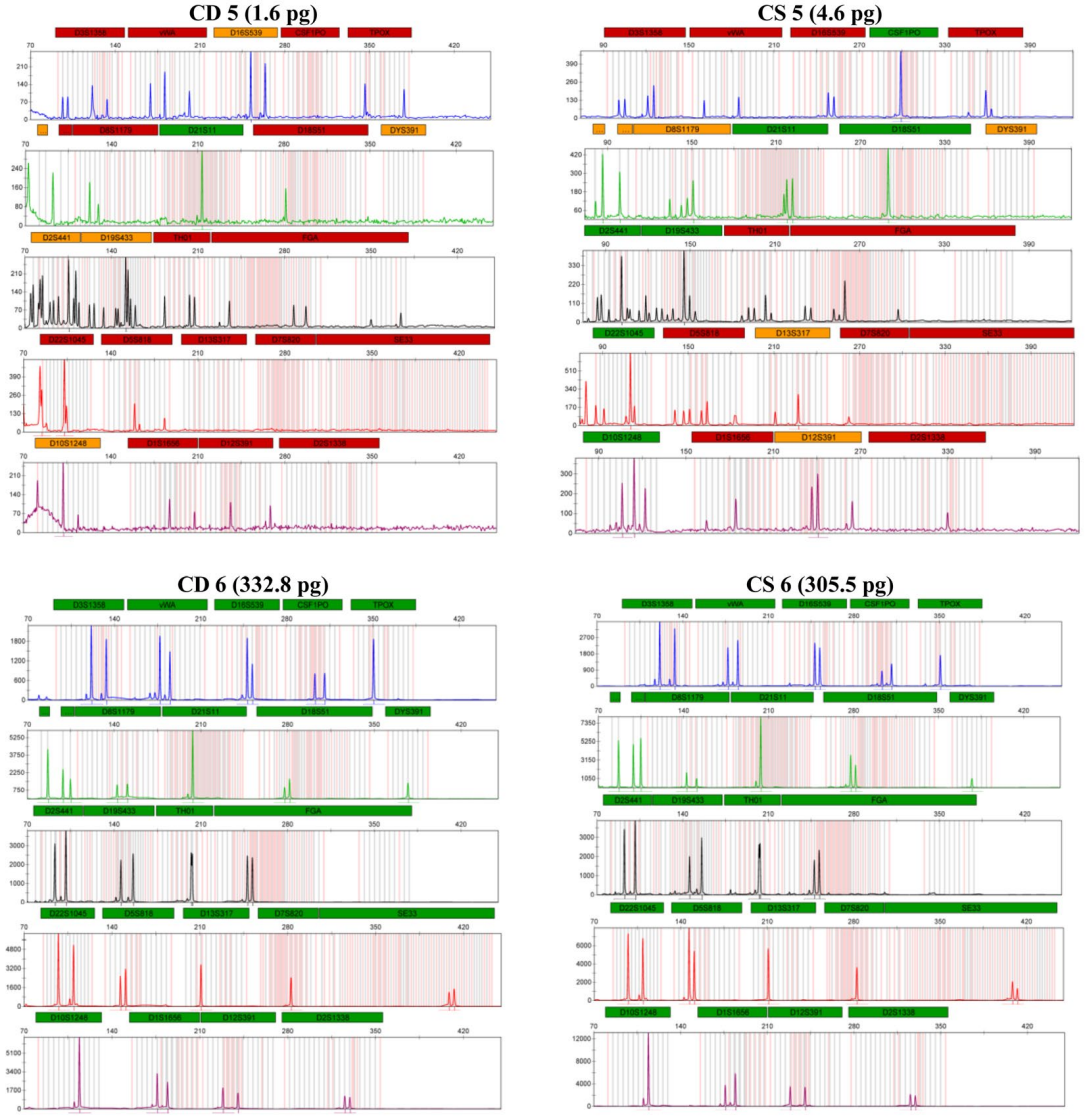
Autosomal DNA profiles developed from the swab of knife’s handle (CD 5 and CS 5) and cuttings from the cigarette butt (CD 6 and CS 6). CD 5 and CD 6 represent the profile from the sample extracted using Casework Direct Kit, Custom. CS 5 and CS 6 represents the profile from the sample extracted using the Maxwell 16 Instrument with the DNA IQ Casework Pro Kit. Samples were quantified using the PowerQuant System on the Applied Biosystems 7500 Real-Time PCR System. Amplification was performed using the Globalfiler Express on a GeneAmp 9700. One microliter of amplified product was electrophoresed on an Applied Biosystems 3500xL instrument using a 1.2 kv, 24-second injection.

**Figure 6:**
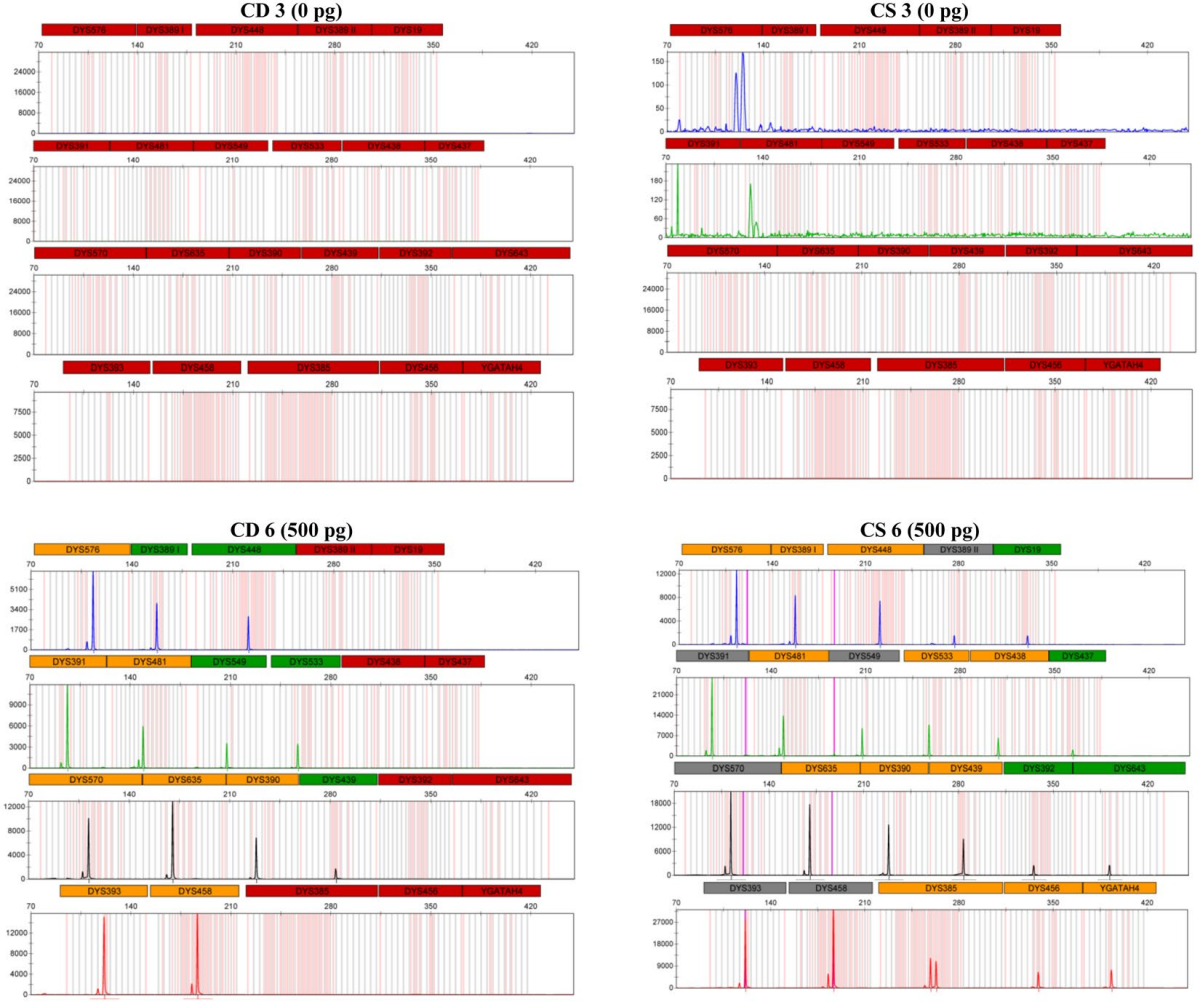
Male DNA profiles developed from the swab of knife’s handle (CD 3 and CS 3) and cuttings from the cigarette butt (CD 6 and CS 6). CD 3 and CD 6 represent the profile from the sample extracted using Casework Direct Kit, Custom. CS 3 and CS 6 represents the profile from the sample extracted using the Maxwell 16 Instrument with the DNA IQ Casework Pro Kit. Samples were quantified using the PowerQuant System on the Applied Biosystems 7500 Real-Time PCR System. Amplification was performed using the PowerPlex Y23 System on a GeneAmp 9700. One microliter of amplified product was electrophoresed on an Applied Biosystems 3500xL instrument using a 1.2 kv, 24-second injection.

The proportions of autosomal and male DNA extracted using Casework Direct Kit, Custom and Maxwell 16 DNA IQ Casework Pro Kit are shown in Table 1 and Figs 1 and 2. We observed relatively better quality and quantity of DNA extracted using Maxwell 16 DNA IQ Casework Pro Kit than the ones obtained using Casework Direct Kit, Custom; see later on number of STR allele calls recorded for both analytical methods. In some cases, several autosomal and male DNA were only detected in casework samples (CS 2 and CS 4 for autosomal DNA and CS 2 and CS 5 for male DNA) extracted using Maxwell 16 DNA IQ Casework Pro Kit DNA (see Table 1; Figs 1 and 2). However, there is no IPC Cq shift flag which indicates both extraction kits are equally good to extract inhibitor free DNA (Table 1). Our results demonstrated that DNA extraction from crime scene samples can be challenging (see Table 1). The presence of biological cells in crime scene samples is normally limited and DNA in the sample may be degraded due to exposure to adverse environmental conditions. In this regard, only a few of the DNA templates extracted from the crime scene and mock casework samples using Maxwell 16 System DNA IQ Kit or Casework Direct Kit, Custom exceeded the require threshold values for reliable autosomal and Y chromosome STR profiling using Globalfiler Express and PowerPlex Y23. Referring to previous studies^16,17^, 125 pg of DNA are required in order to get consistent 100% allele calling with both analytical methods. Here, only about half of the crime scene and mock casework samples (Table 1) extracted using either extraction kit exceeded 125 pg DNA yield. In other words, we anticipated that only half of the samples would produce full profiles. Indeed, from Figs 3 and 4 one can see that when the autosomal and Y DNA input for STR analysis is more than ~125 pg, we did observe mostly 100% allele calling. Thus, full autosomal (6 versus 4) and Y chromosome (6 versus 0) STR allele calls were higher for DNA templates extracted using Maxwell 16 System DNA IQ Kit than using Casework Direct Kit, Custom (Figs 3 and 4 and refer Table 1 for average percentage of allele calling for autosomal and Y chromosome STR). In this validation study, STR allele dropout (see Table 1; Figs 3 and 4) was noted for several samples (e.g. refer CD 8 for autosomal and Y chromosome STR allele calls), with DNA input of less than 125 pg. Nonetheless, autosomal STR peaks (even as large as ~400 RFU) were detected in several samples with low DNA input (<10 pg), but these differ between samples extracted using Casework Direct Kit and Maxwell 16 DNA IQ Casework Pro Kit (see Figs 5 and 6) and can be considered as artefacts. Multiple peaks were observed for sample CD5 and CS5 in Fig. 5. This is expected as CD 5 and CS 5 samples were obtained by swabbing door knob and most likely to have low quality, contaminated and degraded DNA from multiple source of contributors. Contamination of the reagents or inadvertent DNA transfer between samples can be excluded as the cause for multiple peaks observed for CD 5 and CS 5 as no DNA and STR profile were observed for our blank swab samples (Table 1). Several other samples were also flagged as degraded and “ski-slope” effects were seen in their electropherograms. Thus, STR amplification using higher amount of good quality DNA input template will give a better STR allele calls for autosomal and Y chromosome STR profiling.

**Table 1:** Quality and quantity of DNA extracted using Casework Direct Kit and Maxwell 16 System Forensic Kit and amount used for autosomal and Y chromosome STR profiling.

Sample ID	qPCR result (ng/µL)		Total Yield (ng)		[Auto]/[D]	[Auto]/[Y]	IPC	DNA input for STR (pg)		Autosomal allele call (%)	Male DNA allele call (%)
	[Auto]	[Y]	Auto DNA	Y DNA				Auto STR	Y-STR		
CD 1/CS 1	0.0002/0.0018	No Cq/No Cq	0.08/0.09	NA/NA	U/1.6	U/NA	−0.44/−0.29	0.5/5.4	0.0/0.0	45.8/83.3	0.0/4.5
CD 2/CS 2	NA/0.0007	No Cq/0.0005	NA/0.035	NA/0.025	NA/U	NA/1.34	−0.29/−0.47	0.0/2.2	0.0/4.1	70.8/25.0	0.0/27.3
CD 3/CS 3	0.0051/0.0645	No Cq/No Cq	1.53/3.225	NA/NA	3.18/3.33	NA/U	−0.53/−0.30	15.4/193.5	0.0/0.0	66.7/91.7	4.5/13.6
CD 4/CS 4	NA/0.0006	No Cq/No Cq	NA/0.03	NA/NA	NA/U	NA/U	−0.51/−0.46	0.0/1.7	0.0/0.0	25.0/45.8	0.0/22.7
CD 5/CS 5	0.0005/0.0015	No Cq/0.0018	0.2/0.075	U/0.09	1.72/1.8	U/0.85	−0.42/−0.18	1.6/4.6	0.0/13.6	50.0/70.8	22.7/40.9
CD 6/CS 6	0.1109/0.1018	0.0864/0.0746	33.27/5.09	25.92/3.73	2.38/2.93	1.28/1.37	−0.16/−0.15	332.8/305.5	500.0/500.0	100/100	68.2/100
CD 7/CS 7	0.0344/0.0806	0.035/0.0652	10.32/4.03	10.5/3.26	1.84/2.81	0.98/1.24	−0.47/−0.01	103.2/241.9	262.5/488.7	100/100	72.7/100
CD 8/CS 8	0.0181/0.5395	0.0162/0.5063	5.43/26.975	4.86/25.315	3.2/1.85	1.12/1.07	−0.05/−0.14	54.2/1618.6	121.3/500.0	8.3/100	13.6/100
CD 9/CS 9	0.0645/0.2869	0.0289/0.2138	19.35/14.345	8.67/10.69	2.54/2.4	2.23/1.34	−0.4/−0.35	193.6/860.8	216.7/500.0	95.8/100	86.4/100
CD 10/CS 10	0.0739/0.0634	0.0702/0.0597	29.56/3.17	28.08/2.985	1.38/2.14	1.05/1.06	−0.23/−0.12	221.6/190.2	500.0/447.4	100/100	59.1/100
CD 11/CS 11	0.0074/0.0546	0.0053/0.053	2.96/2.73	2.12/2.65	2.35/2.21	1.4/1.03	−0.54/−0.30	22.3/163.9	39.9/397.8	95.8/100	54.5/100
CD 12/CS 12	19.2819/18.902	No Cq/No Cq	7712.76/945.1	NA/NA	1.45/1.31	U/U	0.03/−0.04	500.0/540.0	0.0/0.0	100/100	0.0/0.0
								Average allele call (%)	78.0/92.4	34.7/64.5	

[Auto] – qPCR concentration results of autosomal DNA, [Y] – qPCR concentration results of male DNA, [Auto]/[D] – autosomal STR degradation, [Auto]/[Y] - mixture of female and male DNA, IPC - internal positive control, U – undetermined and No Cq – no cycle quantification value. Values for the two blank swab samples were not included as no DNA and STR allele were observed.

It is interesting to note that quality and number of STR allele calls amplified using Globalfiler Express (Applied Biosystem, Country) and PowerPlex Y23 System both reflect the values given by the PowerQuant assay system (see Table 2; Figs 3 and 4). Our study thus confirms the utility of the PowerQuant System in determine the concentration, purity and mixture of autosomal and male DNA. For example, degradation flag and autosomal/male DNA concentration given by PowerQuant System did predict the quality (e.g. ski-slope effects and artefacts) and quantity (number of STR allele calls) of STR data (e.g. see Table 2; Figs 2–6). This is particularly important for forensic practitioners as the PowerQuant System provides reliable prospective pointers for subsequent analyses; i.e. in autosomal and/or Y-chromosome STR profiling. Therefore, integration of efficient DNA extraction protocols and powerful DNA quantitation methods such as the ones tested here speed up sample processing, reduce inhibitor content, increase DNA recovery from trace evidence and eliminate sample which is unlikely to return informative STR profiles. This should, in turn save time and reduce queues and operational cost. However, our results should still be interpreted with caution as samples used for this study were taken from only a limited numbers of crime scenes. These may, or may not, reflect the actual capability of both extraction kits. Also, there is no way to produce exactly identical pairs of test samples at crime scenes for processing with the two extraction kits which we have examined here (Casework Direct Kit, Custom and Maxwell 16 DNA IQ Casework Pro Kit). In addition, we were also unable to know in advance about the actual presence and absence of DNA in crime scene samples. Nonetheless, data obtained from mock casework and buccal cell samples do provide good comparative data in our test extractions using Casework Direct Kit, Custom, versus Maxwell 16 System DNA IQ kit.

**Table 2:** List of 12 samples used in this assessment study that consist of 8 casework samples, 3 mock samples (prepared in-house via swabbing procedure) and 1 buccal swab sample from a female donor.

DNA source	Sample type	Sample category	Sample ID	
			Casework Direct Kit	Maxwell 16 System DNA
Knife	Swab	Casework	CD 1	CS 1
Hair without follicle	Cutting	Casework	CD 2	CS 2
Plastic straw	Cutting	Casework	CD 3	CS 3
Blood on floor	Swab	Casework	CD 4	CS 4
Door knob	Swab	Mock casework	CD 5	CS 5
Cigarette butt 1	Cutting	Casework	CD 6	CS 6
Cigarette butt 2	Cutting	Casework	CD 7	CS 7
Tissue clot	Cutting	Casework	CD 8	CS 8
Latex glove	Cutting	Casework	CD 9	CS 9
Computer mouse	Swab	Mock casework	CD 10	CS 10
Watch	Swab	Mock casework	CD 11	CS 11
Female buccal cell	Swab	Negative control for male DNA	CD 12	CS 12

## Conclusion

Overall, we can conclude that the Casework Direct Kit and Maxwell 16 DNA IQ Casework Pro Kit have more or less equal capability to extract inhibitor free DNA, but that the latter produces slightly better quality and quantity of DNA templates and subsequently higher numbers of STR allele calls for autosomal and Y-STR analyses (Table 1; Figs 1–6). Our comparative results for the two DNA extraction kits reported here should also be interpreted in a larger context. This includes considering the nature and amount of casework and reference samples currently analysed by forensic DNA laboratories worldwide and the costs associated with DNA profiling. The Casework Direct Kit, Custom could potentially be used for selected analyses. It can easily give comparable results to the Maxwell 16 System DNA IQ Kit for buccal cell extraction (see Table 1 for quality and quantity of extracted DNA and number of STR allele calls). This means, the Casework Direct Kit, Custom is the quicker and cheaper option for extraction of good, clean and high DNA content and might best be used for extraction of all reference samples (e.g. blood or buccal cell samples from suspects, detainees and drug dependents or sample taken for paternity testing). Such reference DNA samples typically come from buccal swabs or freshly drawn blood. So, in general, they can confidently be expected to have a high nucleic acid content and to be inhibitor-free. In our opinion, the two extraction kits examined here together with PowerQuant System should best be used on case-by-case basis in forensic DNA laboratories. Adopting this strategy should ultimately save turnaround time and reduce overall costs associated with routine DNA profiling.

## Materials and Methods

### Samples

Eight casework samples were collected by the Crime Scene Investigation Unit (D10), the Selangor State Police Contingent, Malaysia while three mock casework samples were prepared in-house by swabbing a door knob, a computer mouse and a male’s watch. A buccal swab sample was taken with informed consent from a female donor for a voluntary index of Forensic DNA Databank of Malaysia (section 14 DNA Identification Regulations Act 2012) and was also used as a negative control for male DNA to complete this validation study (Table 1). These samples were prepared in duplicate (i.e. 12 × 2 = 24 samples in total) and each set of 12 were subjected to DNA extraction using Casework Direct Kit, Custom or Maxwell 16 System DNA IQ Casework Pro Kit (labelled as sets CD and CS respectively). This work was conducted as part of the validation study and quality assurance (ISO/IEC 17025:2005) assessment for DNA Databank Division (D13), Royal Malaysia Police Forensic Laboratory (RMPFL) which includes testing new technologies for DNA extraction and STR (autosomal, Y and X chromosomes) profiling. All methods were carried out in accordance with Malaysian DNA Identification Act 2009 and DNA Identification Regulations Act 2012 and all experimental protocols were approved by DNA Databank Division (D13), Criminal Investigation Department, Royal Malaysia Police.

### DNA extraction and quantitation

Genomic DNA was extracted from the prepared set of samples using either the Casework Direct Kit, Custom or the Maxwell 16 System DNA IQ Casework Pro Kit, each according to the manufacturer’s protocols (Promega Corporation, Madison, USA) as previously described^18,19^. Amplification-ready lysate from Casework Direct Kit, Custom and purified DNA from Maxwell 16 System were then quantified by PowerQuant System on an ABI PRISM 7500 Real-Time PCR System (Applied Biosystems, USA) following the methodology established by Promega Corporation (Madison, USA) and those reported in the earlier studies^20–22^. A total of 26 reactions were prepared; the 12 duplicate DNA samples obtained using Casework Direct Kit, Custom or the Maxwell 16 System DNA IQ™ Casework Pro Kit and two blank swab samples as no-template controls (NTC). The reaction mixture consisted of amplification grade water, PowerQuant 2X Master Mix and a mixture of PowerQuant 20X Primer/Probe/IPC Mix. The PowerQuant System quantifies DNA content, determines the male/female DNA ratio, estimates the presence of PCR inhibitors and assesses degradation levels in the tested DNA samples. An [Auto]/[Y] ratio greater than two was taken to indicate for a mixture of female and male DNA while an [Auto]/[D] ratio greater than two was used as a signal for degraded DNA. In contrast, the presence of PCR inhibitors was indicated by Internal Positive Control (IPC), Cq values greater than 0.3.

### STR profiling

The proportions of autosomal and Y chromosome STR loci were amplified using Globalfiler Express (Applied Biosystem, USA) and PowerPlex Y23 System (Promega Corporation, Madison USA) on GeneAmp PCR System 9700 Thermal Cycler (Life Technologies, Foster City, CA). Standard reaction mixture and thermal cycling conditions recommended by the manufacturers were followed. Amplified products were injected at 1.2 kV, 24-s and separated by capillary electrophoresis containing POP-4 Polymer (Life Technologies, CA, USA) on the 3500xL Genetic Analyzer (Applied Biosystems, USA). The capillary electrophoresis results were determined by GeneMapper ID-X version 1.4 software (Life Technologies, USA) with 250 relative fluorescence unit (RFU) set as the peak detection threshold for STR allele calls. The threshold was set during RMP DNA Lab internal validation for both kits. These values are considered as both analytical and stochastic threshold to avoid false allele calling.
